# Edge-colored directed subgraph enumeration on the connectome

**DOI:** 10.1038/s41598-022-15027-7

**Published:** 2022-07-05

**Authors:** Brian Matejek, Donglai Wei, Tianyi Chen, Charalampos E. Tsourakakis, Michael Mitzenmacher, Hanspeter Pfister

**Affiliations:** 1 John A. Paulson School of Engineering and Applied Sciences, Harvard University, Cambridge, MA USA; 2 Computer Science Laboratory, SRI International, Washington, DC USA; 3 Department of Computer Science, Boston College, Chestnut Hill, MA USA; 4 Department of Computer Science, Boston University, Boston, MA USA; 5 ISI Foundation, Turin, Italy

**Keywords:** Neural circuits, Neuronal physiology

## Abstract

Following significant advances in image acquisition, synapse detection, and neuronal segmentation in connectomics, researchers have extracted an increasingly diverse set of wiring diagrams from brain tissue. Neuroscientists frequently represent these wiring diagrams as graphs with nodes corresponding to a single neuron and edges indicating synaptic connectivity. The edges can contain “colors” or “labels”, indicating excitatory versus inhibitory connections, among other things. By representing the wiring diagram as a graph, we can begin to identify motifs, the frequently occurring subgraphs that correspond to specific biological functions. Most analyses on these wiring diagrams have focused on hypothesized motifs—those we expect to find. However, one of the goals of connectomics is to identify biologically-significant motifs that we did not previously hypothesize. To identify these structures, we need large-scale subgraph enumeration to find the frequencies of all unique motifs. Exact subgraph enumeration is a computationally expensive task, particularly in the edge-dense wiring diagrams. Furthermore, most existing methods do not differentiate between types of edges which can significantly affect the function of a motif. We propose a parallel, general-purpose subgraph enumeration strategy to count motifs in the connectome. Next, we introduce a divide-and-conquer community-based subgraph enumeration strategy that allows for enumeration per brain region. Lastly, we allow for differentiation of edges by types to better reflect the underlying biological properties of the graph. We demonstrate our results on eleven connectomes and publish for future analyses extensive overviews for the 26 trillion subgraphs enumerated that required approximately 9.25 years of computation time.

## Introduction

After more than a dozen years of tedious image acquisition and manual reconstruction, *Caenorhabditis elegans* (*C. elegans*) became the first species to have a nearly complete mapping of its neuronal wiring diagram in 1986^1^. This first connectome contained 302 neurons and approximately 5000 chemical synapses. For over twenty more years^2^, and continuing still^3,4^, a significant amount of research has dissected the connectome of *C. elegans*, improving its accuracy and gleaning additional insights. Building on these successes, recent rapid advancements in image acquisition techniques^5–7^ paired with automatic neurite segmentation^8,9^ and synapse prediction^10,11^ methods have enabled the extraction of more complex partial connectomes from more sophisticated species with nearly two and three orders of magnitude more neurons and synapses, respectively^12^. As these automated processes further improve, requiring less human correction and verification, we can expect more diverse animal connectomes at an even larger scale^13–16^.

**Figure 1:**
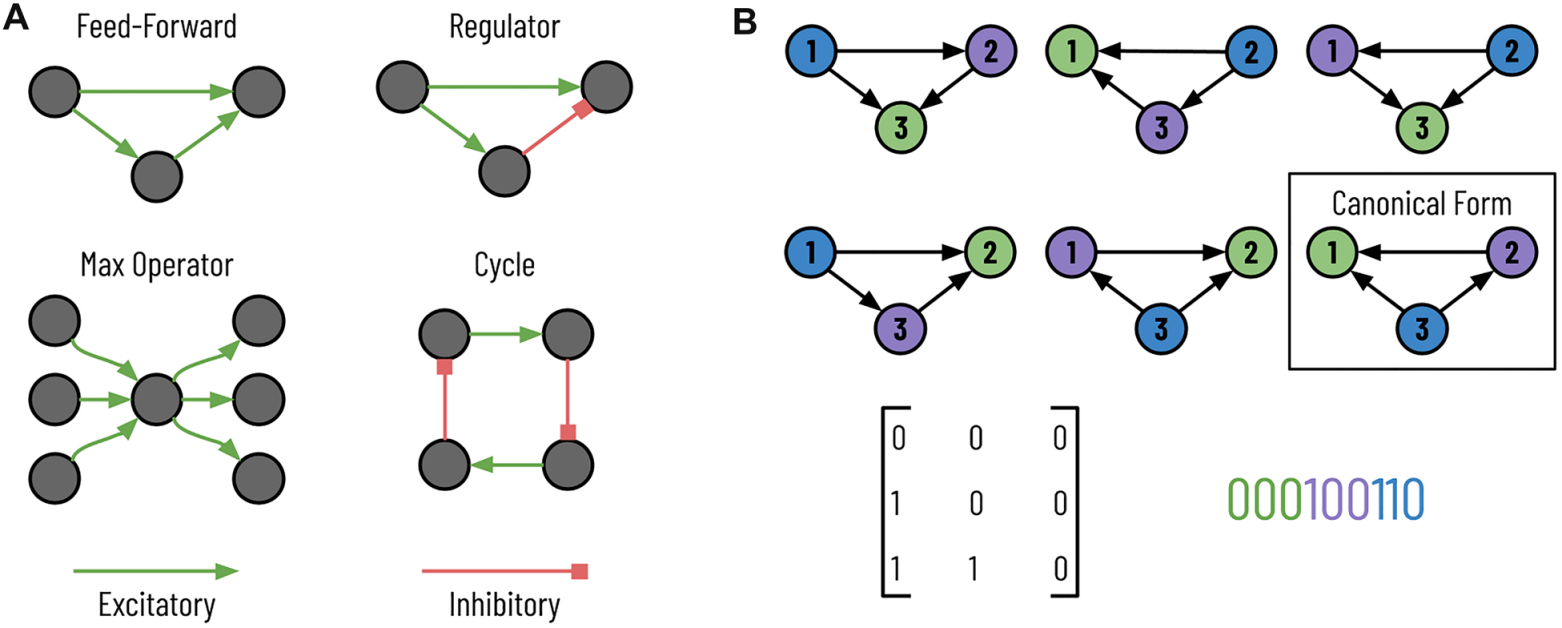
Motifs in the wiring diagram. (**A**) These four subgraphs that appear in the connectome have specific biological functions. Although the biological significance of these motifs was previously hypothesized, enumerating all subgraphs can identify additional essential motifs. (**B**) Here, we see six isomorphic subgraphs of size 3. The node colors do not represent labels but rather correspond to the bijection that preserves edges. The boxed subgraph is the canonical form where the string (bottom right) corresponding to the adjacency matrix (bottom left) is minimized.

Two significant goals for analyzing connectome structures are to generate more faithful models of the brain and improve artificial intelligence^17–19^. To this end, researchers often represent these connectomes as graphs where vertices represent neurons, and neurons that share a synaptic connection receive a directed weighted edge between the corresponding vertices^12^. Furthermore, the graph’s edges can have labels, which we will refer to as colors. Edge colors indicate a specific connection type, e.g., excitatory or inhibitory. After graph construction, one of the primary goals is to identify motifs, small reoccurring subgraphs that correspond to specific biological functions, hidden within the graph (Fig. 1A). A significant amount of existing literature on motif discovery for these connectomes has focused on finding specific motifs of known biological importance within the wiring diagram^12,20,21^. However, an open goal for exploring the connectome is to identify motifs whose biological significance was previously unknown^21^.

**Figure 2:**
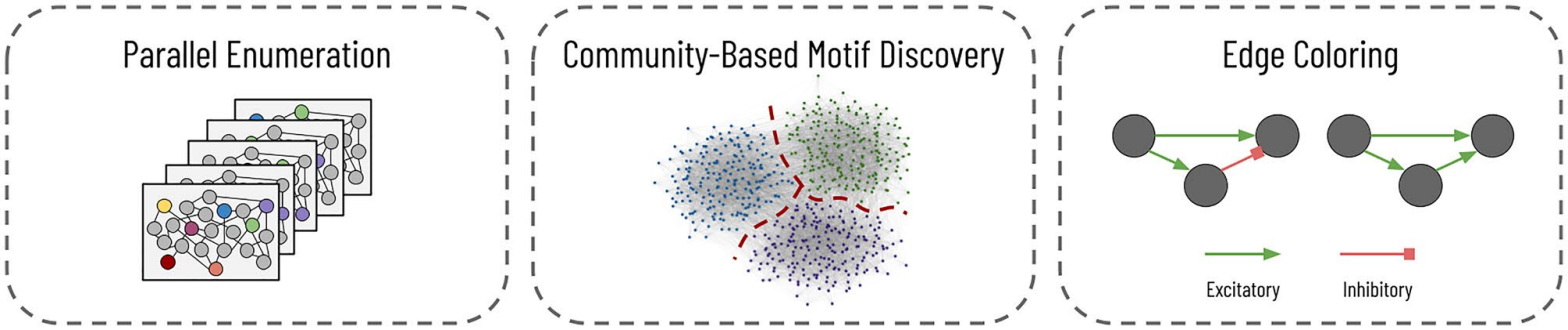
Subgraph enumeration for large-scale connectomes. We propose three improvements on existing subgraph enumeration strategies to improve throughput and better capture the underlying biology. First, we parallelize an existing subgraph enumeration strategy to work on a distributed cluster. For each job, we enumerate a subset of vertices, indicated here by color. Second, we first cluster the vertices into different communities for larger connectomes and perform subgraph enumeration within each community. Lastly, we add edge colors to the graph to match the diversity of the synaptic connections.

One approach to finding these motifs is to enumerate all subgraphs in the wiring diagram to identify those frequently occurring subgraphs. Subgraph enumeration is a computationally expensive task for two reasons. First, the number of subgraphs rapidly expands as the subgraph size *k* increases and the possible set of connected vertices expands. Second, once enumerating a given subgraph, we need to determine its canonical labeling since two subgraphs can have different adjacency matrices and yet belong to the same equivalence class. For example, Fig. 1B shows six subgraphs that have different adjacency matrices and yet belong to the same equivalence class; in the figure, the six subgraphs have an edge-preserving bijection that transforms the vertices from one instance into the other. In Fig. 1B, we color the nodes to help indicate the bijections between the six graphs. Therefore, during subgraph enumeration, we determine the canonical labeling of every subgraph to guarantee that each subgraph uniquely maps to its equivalence class to ensure proper counting. The canonical labeling of a graph is the adjacency matrix of all subgraphs in the equivalence class that has the smallest value when written as a string (Fig. 1B, bottom right). There are no known polynomial-time algorithms to retrieve the canonical labeling from a subgraph^22^. Unfortunately, it is not feasible to count all unique adjacency matrices and determine equivalence classes as a post-processing step. There are $2^{k^2}$ total possible adjacency matrices for a directed graph (noting that self-loops are possible) for a subgraph of size *k*. Compounding these two issues, we must get the canonical labeling for each enumerated subgraph. We propose a parallel subgraph enumeration technique that builds on the existing Kavosh algorithm^23^ (Fig. 2, left). Our method divides the input graph using simple features about the vertices into a set of batch jobs with relatively equal computation times. We reduce the “wall time” over sequential computation needed for one representative dataset by $10 \times$, as we explain further in “Methods”.

Subgraph enumeration becomes infeasible even with parallelization for dense graphs with high average degrees. The size of the available extracted wiring diagrams has dramatically increased in the last decade, and future connectomes will only further increase the number of neurons and synapses^15^. For enumerating larger subgraph sizes, we can first cluster the graph into communities and then perform subgraph enumeration within each community (Fig. 2, center). This two-step divide-and-conquer approach allows us to increase the size of explored subgraphs by significantly reducing the search space. We use an automatic graph clustering algorithm that produces communities of approximately equal size. However, future users could designate communities using existing biological knowledge about the different regions of the brain. Although this approach will miss subgraphs spanning two or more communities, we will still find potentially significant motifs within clusters and later can differentiate motifs between brain regions.

Some existing subgraph enumeration strategies allow one to distinguish vertices by “color” or “label”. Some analyses on the connectome have used these algorithms to distinguish between different types of neurons^20,24,25^. However, few enumeration strategies immediately allow for edge colors without some manipulation. In the wiring diagram, however, edges can correspond to different types of connections with opposite functionalities (e.g., excitatory synapses stimulate and inhibitory synapses suppress) (Fig. 2, right). Identical motif topologies with different connections can produce wildly different neural behavior (Fig. 1A, feed-forward versus regulator). Thus, we need to differentiate between edge types during subgraph enumeration to better match the input graph’s actual biological realities. Although our algorithm allows for the differentiation of either vertex or edge types, we do not discuss vertex coloring since it is already prevalent in the existing literature.

We present four contributions for large-scale subgraph enumeration specifically tailored to connectomic graphs. First, we parallelize an existing subgraph enumeration strategy to significantly reduce the wall time needed to enumerate all subgraphs by balancing the computation time fairly on a distributed computing cluster. Second, we implement a two-step subgraph enumeration strategy that first clusters the input graph into communities and then enumerates subgraphs within each community. We automatically determine these clusters in this implementation; however, this method easily extends to other clustering techniques, including manual labelings based on the underlying biology (e.g., by brain region). Third, we extend the subgraph enumeration task to include edge colors to represent the biologically diverse set of possible connections better. We demonstrate our results on three datasets containing eleven connectomes from two different animal species: *C. elegans* and *Drosophilia*. As our fourth contribution, we provide extensive analysis of the subgraphs found across both species; to facilitate further analyses, we make all data and code publicly available, including the summaries of all unique subgraphs found and their corresponding counts, totaling over 26 trillion subgraphs requiring 9.25 years of computation time.

## Related work

Subgraph enumeration tasks typically fall into two categories: subgraph-centric, also referred to as motif-centric, or network-centric^26^. Subgraph-centric algorithms take as input a query subgraph and identify all occurrences of that particular subgraph^27–29^. These algorithms can efficiently find all occurrences of a given subgraph and use symmetry-breaking conditions to reduce complexity significantly. However, these algorithms require query subgraphs and do not efficiently enumerate all subgraphs, which may be more beneficial in determining the biological importance of all subgraphs. Network-centric algorithms exhaustively enumerate all subgraphs in a given network for a given subgraph size^23,30,31^. These methods enumerate each subgraph once and determine its canonical labeling using Nauty or a similar tool^22^. In this way, these algorithms incrementally determine the number of occurrences for each unique subgraph. Analytic methods count the total number of occurrences of each subgraph type without enumerating all subgraphs by constructing a matrix of linear equations that encode connectivity information^32,33^. However, computational restrictions limit these methods to $k \le 6$^26^. G-Tries represent a middle approach to network- and motif-centric methods^34,35^. This suite of methods constructs a tree of subgraphs, where the tree leaves represent the complete set of subgraphs to enumerate.

As noted above, complete subgraph enumeration is a computationally expensive task as the number of subgraphs grows quickly as *k* increases. Furthermore, there are no known polynomial-time algorithms for producing the canonical labeling of a general subgraph^22^. Approximate counting algorithms reduce the search space by traversing to a new vertex with a fixed probability^36^ or searching a compressed network^37^, among other solutions. However, these strategies can miss subgraphs, which, although very rare, would seldom appear in a random network, and therefore could indicate a biologically important motif (e.g., a large clique). There is a significant amount of research into distributing computation for exact subgraph enumeration. These methods typically look for asymmetrical search spaces induced at each vertex^38^ or edge^39^.

Early research into motifs in neural wiring diagrams typically focused only on small motifs of two or three neurons^40^. Furthermore, these early discoveries typically concerned a few neuron types in specific regions of the brain. Research into motifs in the wiring diagram of *C. elegans* has focused primarily on small motifs between four or fewer neurons^2,25^. Varshney et al. found similar motifs in the *C. elegans* worm as to those found in the mammalian neocortex^2,40^. Cook et al. compare the relative frequency of all possible subgraphs of sizes two and three between the two specific sexes of the *C. elegans* worm^3^. Scheffer et al. consider both small and large motifs of the fruit fly *Drosophila melanogaster*, with attention to neuron type^20^. The authors confirm some of the traditional results from previous works for small motifs, such as the over-representation of reciprocal connections^20,41^. Others have also observed these frequently occurring motifs in the connectomes of mammals and worms. Scheffer et al. also search for families of large motifs, such as large cliques where all possible edge connections are present^20^. However, in both the small and large cases, the authors focus primarily on motifs previously theorized to be critical instead of exploring all possible subgraphs to find new motifs. More recently, Matelsky et al. published DotMotif, a motif-centric connectome subgraph search and query tool that allows for node and edge attribute constraints^42^.

## Results

This section provides summaries and insights on the subgraphs in these connectomes and quantitative statistics on the number of subgraphs and the computation time. To encourage further analyses by others in the field, we provide complete summaries of all subgraph counts for all connectomes. These summaries cover over 26 trillion subgraphs over the eleven connectomes and required over 9.25 years of computation time.

### Datasets

**Table 1:** Connectome datasets. We enumerate subgraphs on eleven connectomes from two different species. Two of the adult *C. elegans* connectomes also contain end-organs and muscles.

Species	Age	Sex	Neurons	Edges	Edge types
*Caenorhabditis elegans*	Adult	Hermaphrodite	473	6897	Chemical/electrical/both
*Caenorhabditis elegans*	Adult	Male	598	7725	Chemical/electrical/both
*Caenorhabditis elegans*	0 h	Hermaphrodite	225	775	N/A
*Caenorhabditis elegans*	5 h	Hermaphrodite	225	986	N/A
*Caenorhabditis elegans*	8 h	Hermaphrodite	225	1006	N/A
*Caenorhabditis elegans*	16 h	Hermaphrodite	225	1101	N/A
*Caenorhabditis elegans*	23 h	Hermaphrodite	225	1504	N/A
*Caenorhabditis elegans*	27 h	Hermaphrodite	225	1524	N/A
*Caenorhabditis elegans*	Adult (50 h)	Hermaphrodite	225	2193	N/A
*Caenorhabditis elegans*	Adult (50 h)	Hermaphrodite	225	2189	N/A
*Drosophila melanogaster*	Adult	Female	21,739	841,720	Moderate/strong

We perform motif discovery on three classes of datasets containing eleven connectomes, ten from *C. elegans*, and one from *Drosophila melanogaster*. Table 1 summarizes each of the eleven connectomes, which are all publicly available.

Our first two datasets contain one complete connectome from each of the two biological sexes of *C. elegans*: hermaphrodite and male. The two sexes have 302 and 385 neurons for the hermaphrodite and male, respectively. These two datasets also include muscles, end-organs, and glial cells leading to 473 and 598 nodes in the graph for the two sexes. Cook et al. produced the first male connectome and provided an analysis of the differences between the two sexes^3^. In their analyses, they focus on subgraphs of sizes two and three only. We extend these analyses by exploring larger subgraphs than initially considered. We also enumerate all subgraphs with three possible edge colors: chemical synaptic connections, gap junction (electrical), or both. We refer to the male and hermaphrodite connectomes from this dataset as *C. elegans AM* and *C. elegans AH* for *adult male* and *adult hermaphrodite*, respectively.

Our next eight datasets come from a longitudinal study of the developmental growth of *C. elegans* published by Witvliet et al.^4^. These eight partial connectomes contain 225 neurons, each with increasing numbers of synapses based on the specimen’s age. Six of these connectomes come from adolescent worms, and the oldest two are adults. These datasets only contain chemical synapse information, so there are no unique edge types. Likewise, we refer to these datasets as *C. elegans D[1–8]* with the number indicating the specimen’s relative age.

The *Drosophila* dataset is the largest publicly available proofread connectome with over 21,000 neurons and four million synaptic connections^12^. We define three synaptic connectivity levels between two neurons: weakly, moderately, and strongly connected. Weakly connected neurons share three or fewer synapses, moderately connected neurons share more than three but fewer than ten synapses, and strongly connected neurons share ten or more synapses. Based on the authors’ discussion on the precision of synapse detection^12^, we prune our graph to contain only synaptic connections that are moderately or strongly connected. Removing these edges reduces the number of edges in our graph from 3,550,404 to 841,720. Compared to mammalian brain samples, the EM imagery does not provide enough detail to differentiate excitatory and inhibitory connections^12^. Therefore, our edges do not reflect that distinction.

**Figure 3:**
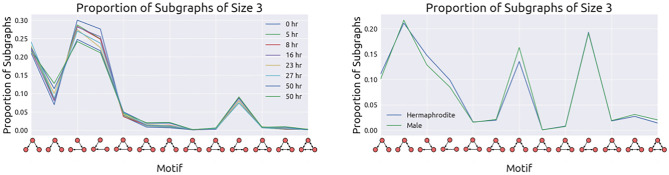
Subgraph distributions of size three. The distributions of subgraphs of size three are similar in the *C. elegans* developmental (left) and the *C. elegans* sexes (right) datasets (cosine similarities > 0.977 and 0.995, respectively). Note that these datasets are not comparable to one another since the datasets differentiating the two sexes include muscles, non-muscle end organs, and glial cells.

### Motifs

**Figure 4:**
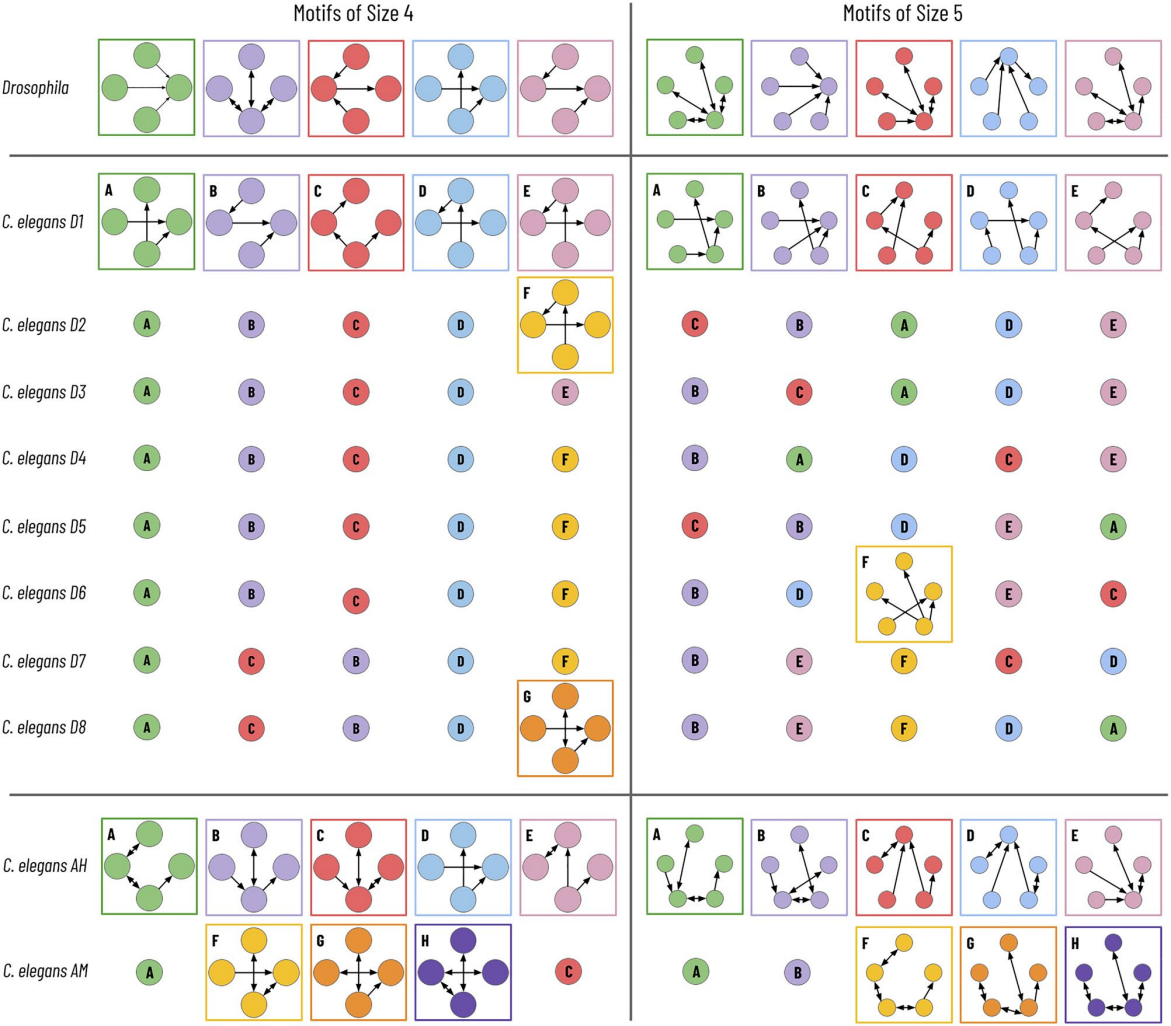
Most frequent motifs of size 4 and 5. Here, we summarize the five most common motifs of sizes four and five for the eleven connectomes. Within each of the three datasets, motifs with the same color and letter designation are identical.

Figure 3 shows the relative proportion of size three motifs for both the developmental growth connectomes and the two sexes of *C. elegans*. The distribution of subgraphs between the specimens in each grouping of datasets is remarkably similar for both subgraphs of size three with a minimum cosine similarity of 0.977 for the developmental series and a cosine similarity of 0.995 for the sexes comparison. This surprised the authors, as these similarities cannot be explained entirely by the multiple connectomes sharing an abundance of joint edges. For *C. elegans* *AH* and *AM*, the two connectomes share 449 neurons, muscles, end-organs, and glial cells, leaving 24 and 149 unique cells for the hermaphrodite and male, respectively. The two specimens have 3572 equivalent edges among the shared nodes, leaving 3325 and 4153 unique edges for each sex. We cannot compare the developmental series with the two other adult worms for two reasons. First, the developmental growth series are only partially connectomes with only 225 neurons each. Second, *C. elegans* *AH* and *AM* contain muscles and end-organs in addition to all of the neurons in the brain.

We provide a more thorough summary of the five most frequent subgraphs of sizes four and five in Fig. 4. We note that the same motifs frequently appear between specimens in the two longitudinal studies. There are only 7 and 6 unique motifs of sizes 4 and 5 for the developmental series over the entire dataset. However, many (but not all) of these motifs represent a purely linear connection with varying directional and bidirectional edges. Subgraph enumeration cannot determine which motifs occur more frequently than random as that requires a random graphical model to serve as a null hypothesis. We can, in part, explain the higher percentage of reciprocal edges in the male/hermaphrodite longitudinal study from the inclusion of electrical synapses, which allow for a bidirectional flow of current.

### Component analysis

#### Kavosh parallelization

Subgraph enumeration for even moderate *k* on the larger *Drosophila* dataset is simply infeasible without parallel processing. We enumerate all subgraphs of size five on this dataset in under 7 days when running on ${\sim 150}$ CPUs. Without parallelization, that enumeration would take 2.77 years (Table 5). Even on the much smaller *C. elegans* datasets, enumerating subgraphs of size 7 on *C. elegans AH* takes 17.95 days of CPU time. Although the amount of total CPU time is similar using a naïve enumeration ordering as to the ordering described in Algorithm 1, the maximum time spent for a single vertex decreases significantly on the *Drosophila* dataset from 9820.40 to 74.12 s ($13.23 \times$). When running on 250 CPUs, our algorithm reduces the wall time by $11.10 \times$ and the idle CPU time by $47.99 \times$ (Table 2).

**Table 2:** Parallelization computation improvements. We can significantly reduce the wall time required when enumerating subgraphs over a compute cluster by relabeling the vertex indices, as described later in the “Methods”. We reduced the maximum time for enumerating from a root vertex from just under three hours to slightly more than 1 min on the *Drosophila* dataset. The new enumeration order reduces the idle CPU time by over 665 h (97.9% decrease). The distribution of individual times for the naïve method is highly skewed to the right; 5% of nodes account for over 62% of computation time.

	Naïve	Algorithm 1
Mean time	8.21 s	8.54 s
Median time	0.92 s	5.80 s
Maximum time	9820.40 s	74.12 s
Wall time	175.20 min	15.78 min
Idle CPU time	680.44 h	14.18 h

#### Community-based enumeration

A divide-and-conquer approach to subgraph enumeration can significantly reduce the total computation time required on a given dataset. The number of subgraphs found decreases, particularly for larger values of *k* (Table 3). These decreases correspond to subgraphs that span more than one community. In going from one to five communities, the number of subgraphs decreases by 55%, with a similar reduction in total computation time. Although this is a significant decrease in enumerated subgraphs, the relative proportions of the motifs found remain relatively stable, as described by the cosine similarity metric, which compares the distances between the two distributions of motif counts. Despite this reduction, we can still identify significant motifs per community. This trade-off between exhaustive enumeration and quicker processing is a necessity moving forward.

**Table 3:** Divide-and-conquer subgraph enumeration. A divide-and-conquer approach can significantly decrease the total amount of computation time required when enumerating very large connectomes, although at the expense of total subgraphs enumerated. These results come from $k = 4$ on the *Drosophila* dataset.

No. communities	No. subgraphs	Total time	Cosine similarity
1	36,041,949,778	51.54 h	1.0000
5	16,111,511,700	23.49 h	0.9577
10	10,660,308,898	15.66 h	0.9207
15	8,103,662,469	11.37 h	0.8693
20	6,340,790,895	8.85 h	0.8244
25	4,563,678,610	6.41 h	0.7946
30	3,819,932,809	5.39 h	0.7915

Although the *Drosophila* dataset is the largest connectome to date, another most likely will surpass it within the next half-decade. A divide-and-conquer approach can avoid enumerating subgraphs that span multiple communities and focus only on those tightly connected regions of the brain. Furthermore, we believe this methodology extends well to connectomes with predefined communities such as different brain regions. In these instances, one could perform intraspecimen comparisons between the motifs found in different communities.

#### Edge coloring

Adding edge colors increases the amount of computation time required for subgraph enumeration. However, differentiating edges by color is critical for these biological graphs where two edges can have markedly different properties. Table 4 gives a brief overview of the increase in running time when adding edge colors to the connectome. Typically, enumerating subgraphs with edge colors increases execution time by $2-2.5 \times$.

**Table 4:** Edge-coloring increased computation costs. Adding edge colors dramatically increases the running time for subgraph enumeration.

Dataset	No color/edge color				
	3	4	5	6	7
***Drosophila***	566.63/1053.48 s	2.15/4.39 days	2.77/6.12 year	N/A	N/A
***C. elegans AH***	0.37/0.77 s	14.75/31.78 s	727.96/1607.75 s	9.53/21.76 h	17.95/42.48 days
***C. elegans AM***	0.38/0.72 s	13.18/29.61 s	593.57/1296.49 s	7.13/16.38 h	12.54/30.11 days

### Enumerated subgraph dataset

We publish exhaustive summaries of the subgraphs found over the eleven connectomes to encourage further analyses. For *C. elegans* *AH* and *AM* datasets, we found all subgraphs $3 \le k \le 7$ with and without edge colors. For the *C. elegans D[1–8]* datasets, we found all subgraphs $3 \le k \le 7$ (no edge colors available). Finally, for the *Drosophila* dataset, we enumerated subgraphs of $3 \le k \le 5$ with and without edge colors. These datasets contain summaries of over 26 trillion subgraphs across all of the connectomes, which required over 9.25 years of total computation time. We hope that this readily available dataset will encourage additional longitudinal studies across species, sexes, and developmental stages in the future.

### Computational complexity

#### Time

Subgraph enumeration is a computationally expensive task, as we see the number of subgraphs quickly explode in the Drosphila dataset (Table 5). Although enumerating subgraphs of size three takes less than 10 min, the total CPU time required balloons to 2.77 years. Comparatively, we enumerate subgraphs for all of the *C. elegans* connectomes considerably quicker. The most time-intensive enumeration for these specimens was for subgraphs of size seven for the adult hermaphrodite (473 vertices, 6897 edges), requiring 17.95 days computation time. We significantly decrease the wall time required by distributing computation across a compute cluster. For the Drosophila dataset, we compressed the 2.77 years of computation time to less than 7 days using ${\sim 150}$ nodes.

**Table 5:** Computational complexity. The number of subgraphs, and the corresponding amount of time needed for enumeration, greatly increases as *k* increases on large connectomes.

Motif size	No. subgraphs	CPU time
Drosophila		
3	126,610,248	9.44 min
4	36,041,949,778	2.15 days
5	12,522,283,314,604	2.77 year

#### Memory

One of the most significant benefits of the Kavosh algorithm is its memory efficiency^23^. Empirically we found that our parallel implementation requires less than 800 MB of RAM for each generated process for subgraphs with six or fewer vertices. Our methods require more RAM for larger subgraphs, although all processes required less than 3 GB.

## Discussion

Twelve years of onerous work yielded the first connectome in 1986 with 302 neurons and over 5000 synapses. Since then, advancements in image acquisition, neural reconstruction, and synapse detection have significantly reduced the amount of time needed to extract these wiring diagrams. A recent dataset of a fruit fly contains over 21,000 neurons and 800,000 moderate and strong synaptic connections. We expect similar growth in the future as neuroscientists reconstruct brain tissue from even more evolved species such as bumblebees, shrews, and even humans. One goal of extracting these wiring diagrams is to create more faithful models of the brain and advance artificial intelligence. Thus, we will need to identify the motifs in these wiring diagrams that correspond to biologically essential functions.

Subgraph enumeration on these dense connectomes is a computationally expensive process that becomes infeasible even for small wiring diagrams with $< 1000$ nodes. We present a novel subgraph enumeration strategy for large connectomes that enables us to find frequent motifs. We parallelize our method to work across a distributed cluster and, when needed, use a two-step enumeration method that first divides the wiring diagram into communities. Building on previous enumeration strategies, we include methods for differentiating edge types to resemble the underlying biology better. We evaluate our methods on eleven connectomes from two species and provide summaries to the over 26 trillion enumerated subgraphs. Ten of these connectomes come from two existing longitudinal studies on *C. elegans*^3,4^. We hope that our published motifs will encourage further analysis into these eleven connectomes and longitudinal studies across brain regions within a specimen and even across different species. Some motifs are known to occur in high frequency across brain regions and animal species, such as the feed-forward loop and the reciprocal connection^3,20,40^. We hope that publishing enumerated subgraphs in variable wiring diagrams will enable the discovery of other biologically essential motifs that span species and offer additional insights into the brain’s inner workings.

As connectomes continue to increase in the number of neurons and synaptic connections, we will need to explore new ways to enumerate subgraphs efficiently. By dividing the brain into individual regions with shared functionality, we can perform intraspecimen longitudinal studies while significantly reducing the computation time for enumeration. Eventually, however, we may need to consider approximate counting methods that sample subgraphs randomly to produce estimates for the motif counts. Although these methods may miss important yet rare motifs, such as fully connected cliques, they will reduce computational costs while still producing extensive summaries of the frequently occurring subpatterns in the brain.

## Methods

### Connectomes as graphs

We construct a graph *G*(*V*, *E*) from each connectome. Each neuron or cell represented in the connectome corresponds to a single vertex in the graph. Each vertex receives a unique index, although these indices do not contain biological significance. Edges in the graph indicate a synaptic connection between two cells. The edges can receive labels (or colors) that can correspond to synaptic strength (moderate or strong) or connection type (excitatory/inhibitory or chemical/electrical). We use *k* to refer to the size (i.e., the number of vertices) of a subgraph throughout the remaining sections.

### Kavosh subgraph enumeration

We extend the Kavosh algorithm to enumerate all subgraphs in our connectomes^23^. This algorithm is extremely fast and easily parallelizable. The Kavosh algorithm begins with the vertex *v* with the smallest index and enumerates all subgraphs of size *k* for which *v* belongs. The algorithms then updates *v* to be the vertex of the next smallest index, enumerates all subgraphs similarly, and so on. At any point, the algorithm ignores any neighbors of *v* that have a smaller index than *v* to avoid counting the same subgraph multiple times since each subgraph has a unique lowest-indexed vertex. This pruning does not miss subgraphs; every subgraph has a lowest value vertex $v_0$, and the subgraph will be found only during the enumeration rooted at $v_0$. The number of enumerated subgraphs grow quickly (Table 5) as *k* increases. Therefore, we cannot store each subgraph as a tuple of vertices as the disk storage quickly becomes too onerous.

### Kavosh parallelization

Since the Kavosh algorithm enumerates all subgraphs rooted at a given vertex, we can divide subgraph enumeration tasks by vertex. We do not need to worry that this division will overcount individual subgraphs since a subgraph is only enumerated if the root vertex has the smallest index. Therefore, we can spawn off as many enumeration threads as vertices and guarantee complete enumeration with no subgraph duplication. However, given the large number of vertices, it is more practical to group together vertices into batch jobs.

**Figure 5:**
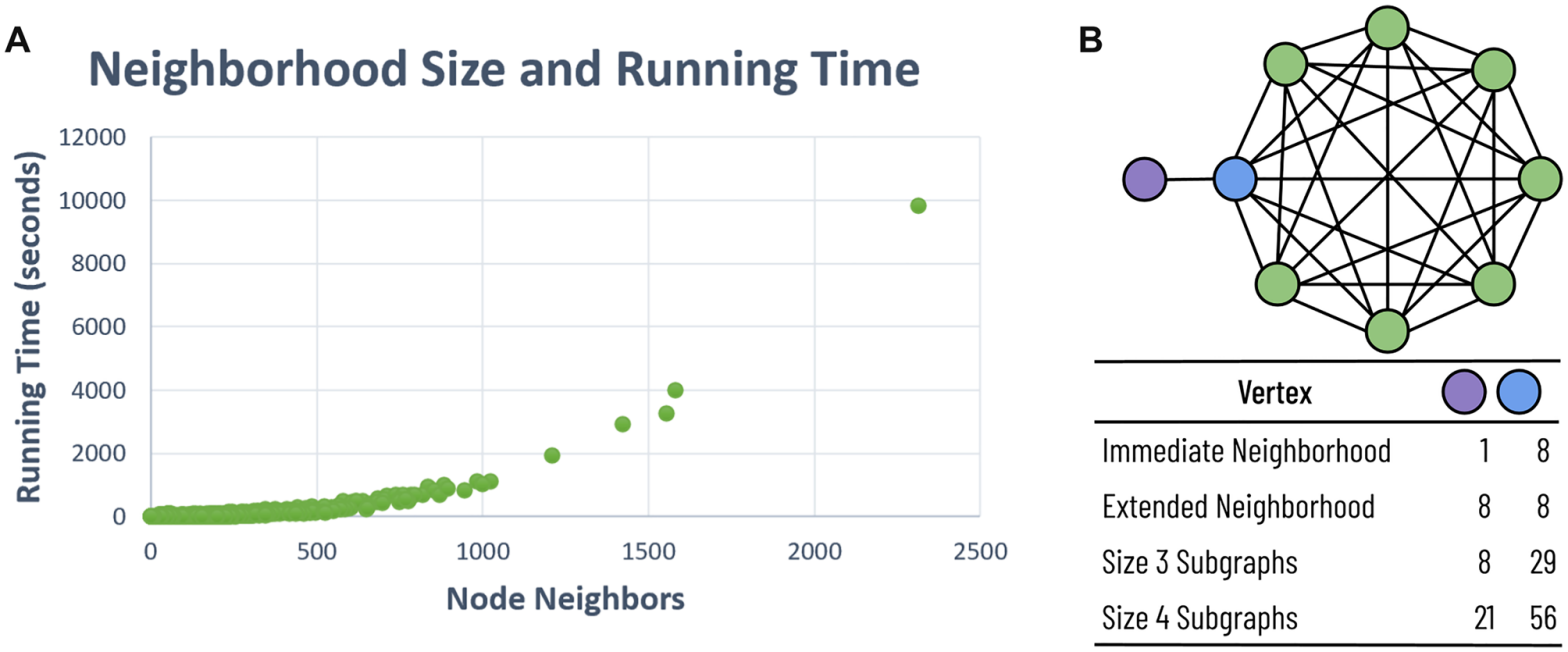
Enumeration times and neighborhood sizes. (**A**) There is a non-linear relationship between the number of neighbors and the running time required. We can reduce running time variance by reducing neighborhood size variance. (**B**) Although immediate neighborhood size is a good indicator of the number of enumerated subgraphs, there is little correlation between the extended neighborhood size and the number of subgraphs.

The running time to enumerate all subgraphs from a given vertex varies greatly (Fig. 5A). In one large-scale connectome with over 20,000 neurons, enumeration of subgraphs of size 4 for some vertices took three or more hours. However, enumeration concluded for 95% of neurons in under 30s. Although such disparities do not significantly influence the total CPU time required, they can drastically alter the “wall time” required when running on a distributed cluster. In most large-scale wiring diagrams, the neuron indices are typically random—an artifact of the automatic reconstruction methods that gradually agglomerate an oversegmentation^8,9^. Even with species with highly stereotyped connectomes, like *C. elegans*, we can merely assign a new “enumeration index” to each vertex that substitutes the given neuron index. Therefore, we selectively choose the “enumeration indices” for each vertex to reduce computation time variance.

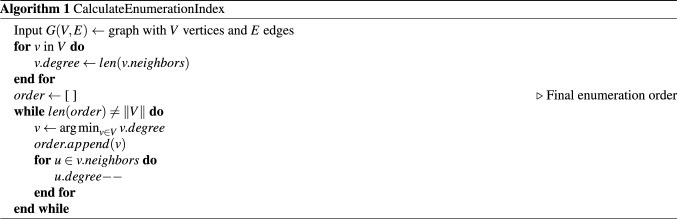



For each vertex *v*, the number of neighbors of *v* with a larger *enumeration index* strongly indicates the number of subgraphs rooted at that vertex, and consequently, the running time required to enumerate from *v* (Fig. 5A). Intuitively, we know that if there are *n* neighbors of *v* with a higher index, at a minimum, there are $$\left( {\begin{array}{c}n\\ k-1\end{array}}\right)$$ subgraphs contained within the immediate neighborhood alone. We only consider the neighborhood of vertices with larger enumeration indices because Kavosh does not enumerate subgraphs rooted at *v* that include vertices with lower-valued indices. When we use the pre-existing neuron indices as the enumeration index, a few unlucky neurons have a low index and an exceptionally high degree. These corresponding vertices dominate the computation time. Therefore, we greedily generate the vertex indices using Algorithm 1. In summary, we assign an enumeration index of 0 to the vertex $v_0$ that has the smallest edge degree. We then decrement $v_0$’s neighbors’ degrees; enumeration from those vertices will not consider subgraphs that include $v_0$. Next, we choose the vertex, $v_1$, with the lowest remaining edge degree, and similarly update the remaining vertices’ neighborhoods. We continue this process until each vertex has an enumeration index.

One might expect that we could gather more information about the possible running times by considering second-order neighborhoods that include neighbors of neighbors. However, we found little correlation between the number of subgraphs to enumerate and second-order neighborhoods. Intuitively, vertices on the periphery of a large clique will have a small first-order neighborhood and a substantial second-order neighborhood (Fig. 5B, purple vertex). However, the number of subgraphs rooted at the peripheral vertex will be significantly less than its neighbor since every subgraph from the peripheral vertex must also include its neighbor. Previous research has noted the existence of over ten such cliques with twenty or more neurons in the *Drosophila* connectome^20^.

### Community-based enumeration

Even with smarter parallelization strategies, enumerating all subgraphs for even $k = 5$ becomes quickly infeasible for large-scale connectome graphs. For a representative connectome with approximately 22,000 neurons and 820,000 edges, subgraph enumeration started at 9.44 min and 2.15 days for motifs of size three and four, respectively, but 2.77 years for motifs of size five (Table 5). Considering that identifying motifs often requires subgraph enumeration on randomized graphs of a similar size, this computational cost becomes overbearing even across a large distributed compute cluster. Therefore, we allow users to cluster the vertices in the graph into communities and perform subgraph enumeration within each community. A downside of using clustering is that we miss enumerating subgraphs that span more than one community. However, we can still identify the frequently occurring motifs within clusters and contrast subgraph counts between different clusters.

For graph clustering, we use the METIS algorithm, which divides an input graph into a predetermined number of clusters^43^. This algorithm has the desirable property of producing relatively evenly sized communities. Other such algorithms provide unequal clusters, which significantly reduces the effectiveness of the divide-and-conquer strategy. Although we use an automatic clustering technique, future analyses could segment the connectomes based on biological priors such as brain regions. With a divide-and-conquer clustering approach, future research could contrast subgraph distributions based on the brain regions themselves.

### Edge coloring

In the innermost loop of subgraph enumeration, we must identify each subgraph’s canonical form to classify a given collection of connected vertices correctly. Although it is not known if the graph isomorphism problem is NP-Complete, there are currently no polynomial time algorithms, and the current best provable complexity is $\exp {\left( \mathscr {O}(\sqrt{n\log {n}})\right) }$^44^. Many motif discovery algorithms use the nauty library for graph isomorphism. This highly optimized library returns the canonical labeling of a vertex-colored graph^45^. However, nauty does not currently support edge colors without some manipulation of the input, requiring an expansion of the graph size to $\mathscr {O}\left( n \log {d}\right)$, where *d* is the number of possible edge colors^22^. Thus, although many motif discovery algorithms allow for vertex colors, they typically do not consider different colored edges. However, different edge types are essential for brain networks and differentiate between excitatory and inhibitory connections and chemical and electrical (gap junction) synapses. Additionally, some pairs of neurons will have multiple pathways between them (e.g., chemical synapses and gap junctions). We can assign a new edge color to indicate neurons that have multiple types of connections. Although the Kavosh algorithm does not natively support edge colors, the nauty documentation briefly discusses how to add edge colors^22^. We modify the enumerated subgraphs before generating the canonical labeling based on the nauty documentation.

### Implementation details

We implement our subgraph enumeration algorithm in C++ and provide a Python wrapper. We use the nauty^22^ library to generate canonical labelings for each enumerated subgraph. We ran timing analysis on a distributed cluster with Intel E5-2695 v2 processors at 2.40 GHz 12 core, with 90 gigabytes of RAM. All code and enumerated subgraph results are publicly available at rhoana.org/subgraph_enumeration.

## Data Availability

We publish the eleven connectomes analyzed on our webpage (rhoana.org/subgraph_enumeration), as well as a link to the motif summaries. Note, the number of nodes and edges on the male/hermaphrodite longitudinal study differ from the original publication of these datasets^3^. Our connectomes come directly from the adjacency matrices in Supplementary Information 5, corrected version July 2020. These datasets contain extrapolated information from other *C. elegans* specimens for cells missing from the reconstruction of Cook et al.
